# 5000 h Multi-Factor Accelerated Aging Test of FRP Made Transmission Tower: Characterization, Thermal Decomposition and Reaction Kinetics Study

**DOI:** 10.3390/polym9050170

**Published:** 2017-05-10

**Authors:** Jianwei Shao, Junhua Wang, Mengjiao Long, Jiangui Li, Yayun Ma

**Affiliations:** 1 School of Electrical Engineering, Wuhan University, Wuhan 430072, China; hitwhshao@whu.edu.cn (J.S.); longmj960@gmail.com (M.L.); 2 School of Mechanical and Electronic Engineering, Wuhan University of Technology, Wuhan 430070, China; 3 Tangshan Power Supply Company of State Grid Jibei Electric Power Company, Tangshan 063000, China; mayayun@whu.edu.cn

**Keywords:** composite transmission tower, multi-factor aging test, aging resistance, mechanical properties, TGA, thermal stability, reaction kinetics, surface morphology

## Abstract

Three kinds of fiber reinforced plastic (FRP) composites, including modified polyurethane resin (LGD), epoxy resin (E44) and modified unsaturated polyester resin (D33) glass-fiber reinforced plastics, were subjected to a 5000 h multi-factor accelerated aging test according to the power industry standard. To examine aging resistance and thermal stability of transmission towers made by these three composites, relevant bending properties, thermogravimetric analysis (TGA) and derivative thermogravimetry (DTG), activation energy, as well as microscopic morphology were revealed. The results showed that for these composites, bending modulus retention rates were higher than 94% under the aging test and that of the LGD was highest. Additionally, the onset degradation temperature, temperature at maximum rate of weight loss and *T*_5%_ reduced at 5000 h, with D33 having highest value and lowest decline rate. The activation energy was calculated with the Bagchi, Coats-Redfern and Broido method, respectively. Although the activation energy of all composites decreased after test, the D33, LGD materials had the highest activation energy which enjoys slight decline. Analysis of the whole experimental results suggested that D33 and LGD composites have good aging resistance, whose basic performance could still perform well after 5000 h aging test, so they can be used to composite towers and applied to engineering practice.

## 1. Introduction

Traditional transmission towers, which are vulnerable to rust or cracking, have the disadvantages of heavy quality, poor durability, short service life expectancy, and are prone to a variety of security risks. With the development of material technology, and continuous improvement in productive technology of fiber reinforced plastic (FRP), the application of composite materials can be achieved in the field of power transmission towers. Their associated technical advantages include: (1) light weight; (2) high strength; (3) temperature adaptability; (4) excellent electric insulation properties; (5) easy to mold; (6) maintainability; and (7) environmental friendly [1]. It can be seen that the composite towers and crossarms have excellent comprehensive performance characterized as a new structure of low carbon, energy saving, environmentally friendly and compliant with aesthetics, representing one of the strategic directions of transmission towers. Therefore, it would be a revolution in the construction of power industry infrastructure to promote the application of composite material towers in the transmission line as long as the material performance can meet requirements.

Aiming at promoting the application of FRP in composite material towers, some institutions and scholars have carried out some preliminary exploration researches. In order to solve the problem of insulator pollution flashover, Hidel Okamoto and Yasuyuki Ikeda worked on the discharge test using FRP tower crossarm, and found that FRP has a good characteristic of arc resistance, which is the earliest mention of the application of FRP in transmission towers [2]. Subsequently, Miller and Hosford proposed the idea of using FRP poles instead of traditional wooden poles and built 75-base FRP poles in areas where vehicles could not reach in order to avoid the problem of hardening maintenance of the wooden poles and enhance the reliability of the line [3]. Awad et al. elaborated the prospect of using the organic material to make transmission towers, and compared the steel with FRP materials in the same tower size from an economic point of view [4]. Major transmission and distribution companies in the USA have showed a strong interest in composite towers. The manufacturing enterprises, including Ebert Composites, Powertrusion Composites, Shakespeare, North Pacific and CTC have actively developed a variety of composite towers products and applied for patents [5,6]. While in Canada, another North American country, the interest in composite towers is not strong, because of the lack of confidence in the anti-ultraviolet aging performance of composite towers. As early as 1960s, Japan carried out the research on the FRP crossarms and found that these crossarms could avoid flashover accidents caused by windage yaw [7]. In China, researches into transmission towers mainly focused on the reliability of truss structure tower at high voltage level. Little research concentrated on alternative materials. In the construction of electric power engineering, FRP is mainly used in high voltage composite insulators and waste gas measurement systems in power plants. There are few relevant reports of using FRP material for manufacturing transmission towers. Only Wuhan NARI Limited Liability Company of State Grid Electric Power Research Institute [8,9] and Wuhan University [10,11,12] carried out some preparatory studies. According to the unified deployment of State Grid Corporation of China (SGCC), pilot projects of composite material towers were carried out in Shandong (35 kV), Tianjin, Hunan, Shanghai, Zhejiang, Beijing, Fujian (110 kV), and Jiangsu (220 kV). Meanwhile, Wuhan NARI conducted basic material performance test, electrical performance test, lightning protection test and calculation.

Previous research made some achievements in composite towers and proved the feasibility and advancement of the composite material used in the transmission towers. However, it should be clearly recognized that the following key problems need to be addressed before composite materials are applied to transmission towers: (1) lack of operational experience. Style, size, mechanical properties and electrical performances are unknown currently; (2) FRP’s anti-aging performance under long-term operation in complex transmission line corridors environment is not clear.

Through the above analysis, it can be seen that the key problem, the lack of long-term anti-aging performance research of FRP towers in the complex transmission corridors, needs to be solved. Without the resolution of this problem, the follow-up researches and application of FRP composite material tower would be greatly restricted. Therefore, a comprehensive assessment of anti-aging properties of composite materials used for towers has been carried out in the School of Electrical Engineering, Wuhan University. Three kinds of FRP materials were selected to carry out a 5000 h multi-factor artificial accelerated aging test according to IEC/TR 62730-2012 for 5000 h aging test [13]. During the aging process, a bending test of the material was carried out to investigate the mechanical properties of the material under aging conditions. Thermal stability was investigated by thermogravimetric analysis. Since the thermal degradation activation energy can directly reflect the thermal stability of the polymer, the kinetic parameters of the reaction were also calculated. Microscopic observation of microstructures was carried out to analyze the microscopic changes of materials. Thus, the long-term safety of the composite material tower can be verified, and the best anti-aging material can be selected.

## 2. Materials and Methods

### 2.1. Materials

Glass fiber is used as a kind of reinforcing material, which has gained an international consensus. The epoxy resin or thermosetting polyurethane resin is mainly used as resin matrix. RS technology company in Canada uses thermosetting polyurethane resin as resin matrix to make composite towers. In China, there are epoxy resins, unsaturated polyester resins and polyurethane resin composite towers as well, among which epoxy resin is in the great majority [14]. According to the previous literature in FRP materials-made composite towers, modified polyurethane resin, epoxy resin and modified unsaturated polyester resin glass-fiber reinforced polymers were selected in this study. The specific information of the samples is shown in Table 1. Composites standard splines were prepared by hand lay-up process, curing 2–3 h at 80 °C after gelling at ordinary temperature. Then an engraving machine (the accuracy is ±0.2 mm) was used to cut the composite materials into standard splines whose size is 120 mm × 15 mm × 4 mm. The standard EWR400 glass fabric was used to prepare 1:1 woven roving glass fiber reinforced polymer composites, whose thickness is 0.4 mm, mass being 400 g/m^2^, moisture content being less than or equal to 0.2. At the same time, three resin matrix samples were prepared using the same method.

### 2.2. Expermental Setup and Procedure

At present, there are no composite material tower aging test standards. However, for HV polymeric insulators, there is a corresponding 5000 h test technical report set by International Electrotechnical Commission (IEC) named IEC/TR 62730-2012 [13]. Considering that the operating environment of the composite tower is identical to that of composite insulators, this technical report can be a useful reference. Based on the multi-factor aging test chamber at Wuhan University, the aging test of the processed composite samples was carried out by referencing to IEC/TR 62730-2012.

The multi-factor aging test chamber is a device for testing the life of composite materials under artificial simulated environmental conditions. The device simulates the effects of solar radiation, rainfall, damp heat, high and low temperature alternation and salt spray environments, which could possibly occur in the natural environment. Through program control, a periodic comprehensive aging environment is manufactured including a variety of factors. This multi-factor aging test chamber is 2.0 m × 2.0 m × 2.0 m with a control screen, where the following technical parameters can be implemented:

(1) Precise humidity control. Humidity range: 30% RH~98% RH. Humidity should reach 98% RH within 20 min. Relative humidity accuracy: +2~3%;
(2) Precise temperature control. Temperature control range: −30~60 °C. Control accuracy tolerance: ±0.5 °C, closed-loop control used;
(3) Rainfall and rainfall regulation system. Rainfall intensity range: 10 ± 5 mm/h~100 ± 20 mm/h.
(4) Salt fog test equipment. Flow rate: 0.4 ± 0.1 kg/m^3^·h. NaCl volume: 2.5~10 kg/m^3^;
(5) UV lamp irradiance: 50–100 W/m^2^;
(6) Voltage and insulation requirement. The high AC voltage is transformed into the chamber through the high voltage bush. The test chamber can withstand 60 kV voltage.

**Figure 1:**
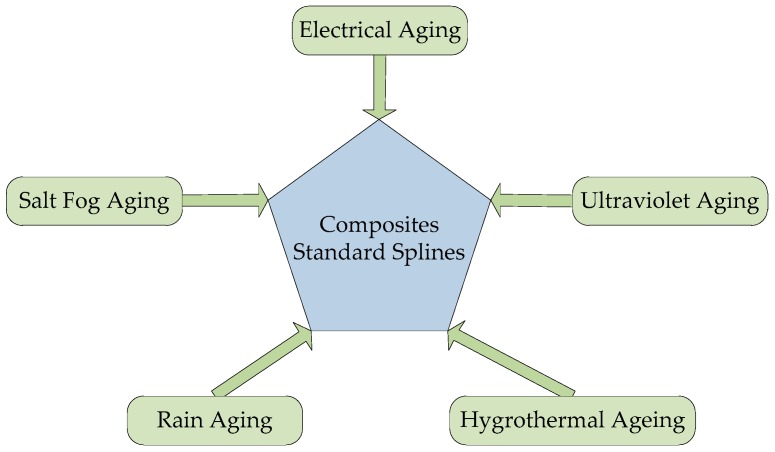
The basic structure of multi-factor aging system.

This study simulated the real operating environment of a transmission tower, including aging factors of rain (about 1.2 L/h), salt fog (7 kg/m^3^), high temperature (50 °C), high humidity (95%), low temperature (−30 °C), ultraviolet rays (100 W/m^2^) and 10 kV electric field and the test time being 5000 h. The basic structure of the whole multi-factor aging system is shown in Figure 1.

The composite’s standard splines were placed in this multi-factor aging test chamber. The accelerated aging test was carried out according to test procedure in Table 2. The aging time was 5000 h. The composite samples and the resin matrix samples (five splines for each material) were taken out every 500 or 1000 h for mechanical performance tests. At 0 and 5000 h, the composite samples (one spline for each composite material) were taken out for thermogravimetric analysis and microscopic analysis.

### 2.3. Charaterization

In this paper, the mechanical property, thermal stability and micro-performance of these three materials were tested at different stages of aging test, to study the aging resistance and high-temperature resistance of materials used for transmission towers. The thermal decomposition reaction kinetics were also analyzed. The combination of these three tests can fully reveal whether the aging resistance of composite towers can meet practical engineering needs during long-term running in practice.

#### 2.3.1. Bending Test

During the aging test, the composite material samples and resin matrix samples were taken from the aging test chamber every 500 h and then the bending modulus was tested according to ISO 14125:1998 [15]. During the test, the temperature remained at 30 °C, the span being 80 mm, and load was 60 N. Each sample was tested five times and the final data value was averaged. At the same time, the bending strength test was also conducted referring to ISO 14125:1998.

#### 2.3.2. Thermogravimetry Analysis

These three kinds of composite samples were subjected to thermogravimetric analysis (TGA) and derivative thermogravimetry (DTG) respectively at the beginning of the aging test (0 h) and the end of the test (5000 h). TGA and DTG of the samples were performed in the atmosphere of nitrogen, using TGA Q500 (TA Instruments, New Castle, DE, USA) V20.2 Build 27. The heating rate was 10 °C/min and the flow rate was 20 mL/min, the temperature ranged from 30 to 650 °C. 

The normal methods to determine activation energy and most possible mechanism functions in thermal analysis kinetics (TAK) are described in this section of this paper.

● Determination of TGA parameters

The onset degradation temperature (OT) was determined by the intersection of tangent at the maximum slope and the extension baseline. The final residue (FR) was taken from ordinate of the final residue in the TGA curve. *T*_5%_ was determined by the temperature at which 5% weight loss occurred, and the temperature at maximum rate of weight loss (DTG_max_) was confirmed according to the peak value of the DTG curve.

● Determination of the Activation Energy

At present, there are few studies on the thermal decomposition kinetics of glass fiber composites, and the thermal decomposition kinetics of different types of glass fiber composites are usually quite different [16,17]. Reference [18] calculated the thermal decomposition kinetic parameters of glass—fiber/epoxy composite based on the multi-step decomposing model of Arrhenius equation and the direct solution method; it proved that the thermal decomposition kinetics parameters obtained by direct solution are correct and effective. Reference [19] studied the thermal decomposition reaction of 4-hydroxypyridine blocked isophorone diisocyanate and analyzed the thermal decomposition reaction kinetics by the Friedman–Reich–Levi (FRL) equation, Flynn-Wall-Ozawa (FWO) equation, and Crane equation. Reference [20] revealed the degradation mechanism of Cellulose tri-stearate and calculated the activation energy by means of the Ozawa method, Coats-Redfern method and Kinssinger method. 

The activation energy *E* of D33, E44 and LGD was analyzed by the integral method (Broido, Coats-Redfern equation) and the differential method (Bagchi equation) [21,22,23].

The fractional conversion can be obtained from the following equation

(1) $$\alpha =(W_{0}-W_{t})/(W_{0}-W_{\infty })$$

The kinetic model function is *f*(α), and *G*(α) represents its integral form. (This article assumes that $f(\alpha )={\left(1-\alpha \right)}^{n}$ firstly, where *n* is the order of reaction, *n* = 1).

Broido equation [21] is written as follows:

(2) $$\ln[G(\alpha )]=\ln\frac{ART_{m}^{2}}{\beta E\exp\left(\frac{2E}{RT_{m}}\right)}+\frac{E}{RT}$$

where *T* is the temperature of derivative curve of TGA; *T*_m_ is the temperature at the point of the maximum weight loss; *R* = 8.3145 J·mol^−1^·K^−1^, referring to the gas constant; β is the heating rate, which can be obtained by β=dT/dt.

E represents the activation energy that can be calculated from the slope of ln[G(α)] versus $\frac{1}{T}$. For f(α)=1−α, E can be calculated from the slope of $\ln\ln(\frac{1}{1-\alpha })$ versus $\frac{1}{T}$.

Coats-Redfern equation [23] is shown as follows:

(3) $$\ln[\frac{G(\alpha )}{T^{2}}]=\ln[\frac{AR}{\beta E}(1-\frac{2RT}{E})]-\frac{E}{RT}$$

Since the first term at the right end of the equation is almost constant, E can be calculated from the slope of $\ln[\frac{G(\alpha )}{T^{2}}]$ versus $\frac{1}{T}$. For f(α)=1−α, it can be calculated from the slope of $\ln[\frac{-\ln(1-\alpha )}{T^{2}}]$ versus $\frac{1}{T}$.

The kinetic parameters obtained by the integral method are in a reaction interval, but for some initial kinetic irregular reactions (e.g., decomposition of polymer), the results gained from this method are inaccurate. The differential method is based on the relationship between the mass loss rate and the temperature, which means that the parameters are obtained from the instantaneous values. So the differential equation is better when we solve the thermodynamic parameters of the composites in this study.

Bagchi equation [22] is written as follows:

(4) $$\ln[\frac{d\alpha /dT}{f(\alpha )[\frac{E(T-T_{0})}{RT^{2}}+1]}]=\ln\frac{A}{\beta }-\frac{E}{RT}$$

*T*_0_ is the onset reaction temperature, which may be defined as the temperature at which α is negligibly small.

The left part of Equation (4) has a linear relationship with $\frac{1}{T}$. For each differential function *f*(α), Equation (4) can be solved by an iterative method. Given any *E*(>0), which can be used to calculate the corresponding value of the left Equation (4) for each data point, and then a new *E* will be derived from the slope using the least squares method. The correct value of *E*, as a new initial value, can be iterated again to get another updated value. After several iterations, the most suitable *E* value will be obtained.

As the result obtained by the integral method may be inaccurate, in this paper, firstly, the differential method is used to calculate the activation energy of thermal decomposition reaction and determine the reaction mechanism function. Then the integral method is used to supplement the conclusion. 

● Determination of the *G*(α)

Firstly, plot the curve of left part of Equations (2)–(4) versus $\frac{1}{T}$. If the function is chosen properly, a nearly straight line can be obtained, implying the selected function *f*(α) or *G*(α) can reflect the specific reaction mechanism. If not, the function form must be re-selected, then kinetic parameters need to be recalculated and plots remapped using trial and error method until the appropriate function is found. The reaction mechanism function can be selected from Table 4 of T.P Bagchi’s paper [22].

#### 2.3.3. Surface Morphology Test

A field emission scanning electron microscope (FESEM) (SIGMA, Carl Zeiss AG, Oberkochen, Germany) was used to examine the surface morphology of the samples. The E44, LGD and D33 composite specimens were cut out from the samples before and after the aging test, cleaned with an ultrasonic cleaner. Small specimens of the samples were cut from the surface of the sample within 3 mm depth. Then the pieces were sprayed before the FESEM scanning.

## 3. Results 

### 3.1. Bending Test

**Figure 2:**
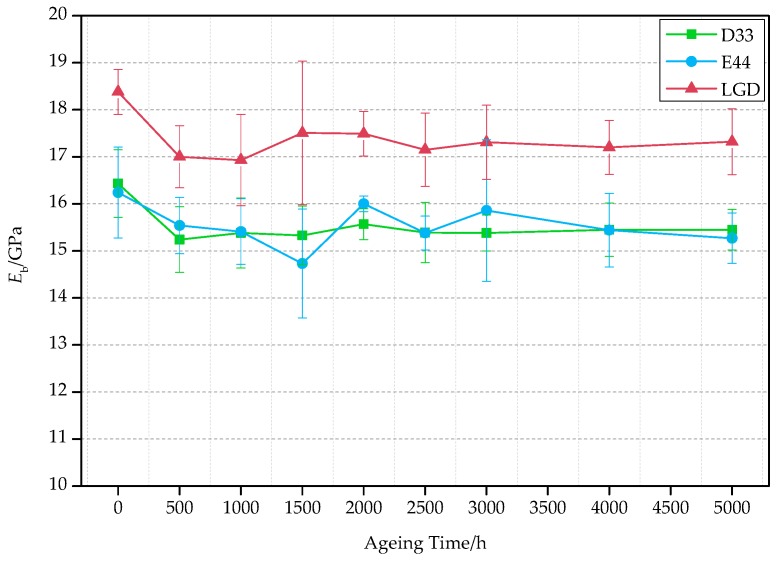
The variation of the bending modulus with time.

Table 3 is a list of the bending modulus and retention rate of the three composites. Figure 2 shows the time-varying bending modulus. Figure 2 presents that the bending modulus of the three materials’ decrease with the multi-factor aging time from 0 to 5000 h, decreased by about 6% at 5000 h. The *E*_b_ of the LGD composite samples is higher than that of the other two materials during the whole aging process by about 2 GPa. The composite samples are not fully cured before the test and the post curing crosslinking reaction occurs at the initial stages of the aging test. The decrease of mechanical properties of materials in the aging process is mainly due to the light, heat, water, oxygen, radiation effects which cause fracture of polymer molecular chain and de-bonding of fiber/matrix interface and lead to the decrease of capacity of matrix and interface load transfer. Additionally, the chemical medium can also damage the structure of glass fiber, which results in the decrease of capacity of fiber-bearing load.

The bending modulus retention rates of the three composite samples are higher than 94% after 5000 h aging test, indicating that the mechanical properties and aging resistance of these composites are excellent as expected.

In this research, the bending modulus of resin samples were also tested at the same time. The results are shown in Table 4. The bending modulus of the resin is only one tenth of that of the composites in Table 3. The addition of glass fibers improves the bending modulus of the resin base. After the 5000 h aging test, the retention rates of LGD and D33 resin matrix are higher than that of composite materials. Due to the destruction of the fiber-matrix interface, the mechanical properties of the composites decrease rapidly than resin matrix. 

Because of the extremely low bending modulus, resins cannot be used for transmission towers. Therefore, in the subsequent experiments, no thermal stability and microscopic analysis of the resin samples are carried out. 

Table 5 is the results of bending strength (*f*) of composites before and after the aging test. As can be seen from Table 5, the bending strength retention rates of these three materials are higher than 90% after 5000 h, and the absolute values are over 400 MPa.

### 3.2. Thermogravimetric Analysis (TGA)

#### 3.2.1. Basic Data from TGA

**Figure 3:**
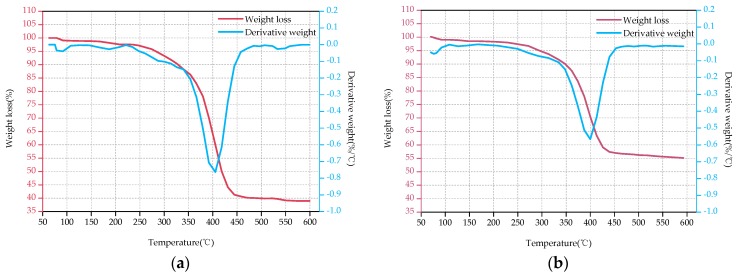
TGA-DTG thermograms of D33 before the aging test (**a**) and after the aging test (**b**).

**Figure 4:**
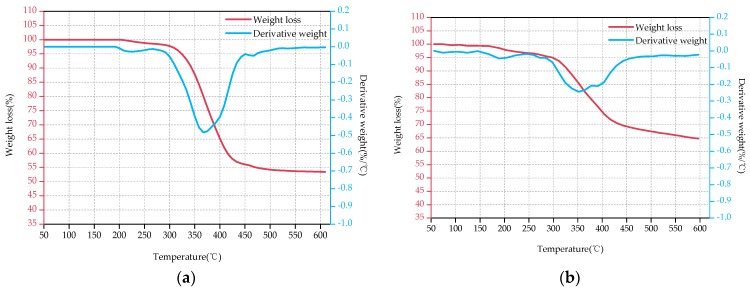
TGA-DTG thermograms of E44 before the aging test (**a**) and after the aging test (**b**).

**Figure 5:**
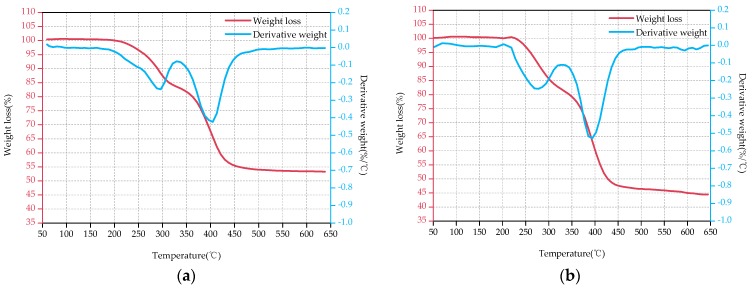
TGA-DTG thermograms of LGD before the aging test (**a**) and after the aging test (**b**).

Figure 3, Figure 4 and Figure 5 show the TG and DTG curves of D33, E44 and LGD composite samples before (a) and after the aging test (b). As for D33 and E44 samples, the DTG curves have only one loss peak (Figure 3 and Figure 4), indicating that decompositions of D33 and E44 composite materials have one step, in other word, only one loss mechanism. It can be considered as the thermal decomposition of resin matrix during the main degradation range. These two types of materials have only a small weight loss (less than 2%) below 250 °C, which is mainly caused by the evaporation of its moisture adsorbed during the aging test. However, two significant weight loss peaks emerge at about 275 and 400 °C in Figure 5. That is, there are at least two different weight loss mechanisms of LGD material. Therefore it is concluded that the two weight-bearing peaks correspond to the thermal decomposition of epoxy resin matrix and residual carbon.

Table 6 displays the data obtained from the TG and DTG curves. As can be seen from Figure 3, Figure 4 and Figure 5 and Table 6, the decomposition temperature OT of the aged samples declines in comparison with the original samples, declining by 1.69%, 10.77% and 1.62% for D33, E44 and LGD composite samples respectively. OT of D33 (354, 348 °C) is higher than that of E44 (325, 290 °C) and LGD (247, 243 °C) during the whole test, and the OT of LGD is the lowest. For DTG_max_, the same conclusion can be drawn. DTG_max_ reduces after aging for all samples, with the magnitude of reduction being 0.25%, 3.29%, and 2.72% respectively for D33, E44 and LGD materials. DTG_max_ values of D33 (401, 400 °C) and LGD (405, 394 °C) are not much different before and after the aging test, which are greater than that of E44 (365, 353 °C). *T*_5%_ of E44 and LGD also has the same trend, 8.02% and 1.52% decrease respectively (except for D33, an increase of 6.83%). Under the continuous multi-stresses, the severely aged composite samples degrade rapidly, leading to the reduction of OT, DTG_max_ and *T*_5%_.

The FR of D33, E44 samples increases with the increase of the aging time and the rate of increase is 41.03% and 20.97% respectively. The residual amount of D33 samples is less than that of E44 (39.0% < 53.4%, 55% < 64.6%) for both the original samples and the aging samples. Reference [24] shows that when the material temperature reaches to a certain level (200~300 °C), the resin matrix begins pyrolysis, with decomposition products of gas and coke. The final pyrolysis products of D33 and E44 samples are the coke, glass fiber and small molecule gas. As the aging time prolongs, the structures of the D33 and E44 samples are aggravated. In the thermogravimetric analysis, the D33 and E44 samples with severe structural damage are sufficiently degraded, resulting in more carbon and volatile gases. The FR of LGD materials decreases with the increase of aging time (53.3% → 44.5%), which may be due to the addition of flame retardants in the material. Flame retardant components will be degraded firstly in the aging process, resulting in the capacity of flame retardants contributing to carbon reduced, so the residual rate decreased [25,26].

From the above analysis of the thermal properties of these samples, it can be obtained that the comprehensive aging will reduce the thermal stability of D33, E44 and LGD composites. From the perspective of OT, DTG_max_ and *T*_5%_, the absolute value of D33 is high during the test, and the rate of degradation after aging is low, which represents a high aging performance and thermal stability. These three parameters show that the greatest drop of thermal stability occurs in E44 splines which have the lowest thermal stability. FR parameters show that, after 5000 h aging, the performance of the three composites decreases to varying degrees with destroyed structures, and the additives are volatile.

The reasons for the decrease of thermal stability are quite complex. The humidity, temperature, rain, salt spray and light environment during the multi-factor aging test may cause the degradation of the thermal stability of composite materials. In addition, the impact of electrical aging on the material is from the outer surface to the inside area, degrading polymers, cutting off macromolecular chain and increasing unsaturated double bonds. 

#### 3.2.2. Calculation of Activation Energy (*E*)

● Differential method

First and foremost, *T*_0_ must be determined in order to calculate the kinetic parameters using Bagchi methods. Table 7 gives the values of *T*_0_ for the three composite’s samples determined from the thermogravimetric analysis data.

**Figure 6:**
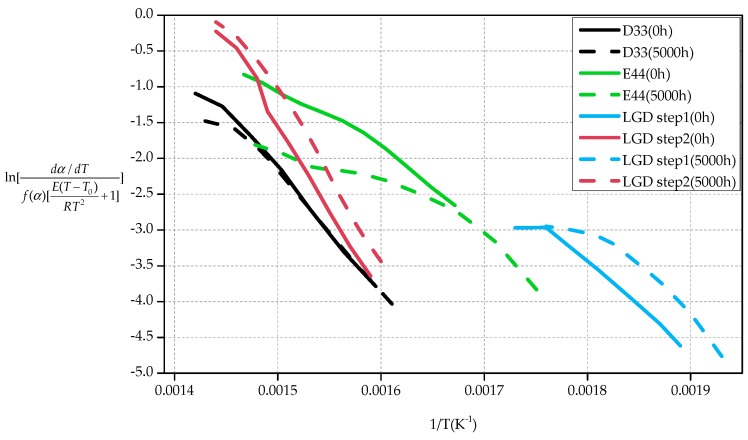
Bagchi plot for determination of *E*.

Table 8 shows the iteration results of D33 and E44 using the Bagchi methods when *f*(α) = 1 − α. The activation energies of D33 before and after the test are 134.35 and 124.38 kJ/mol respectively, decreasing by 7.42%. The activation energies of E51 is 72.75 and 54.53 kJ/mol respectively, decreasing by 25.04%. However, for LGD samples, the results of step1 are nonlinear when *f*(α) = 1 − α. Therefore, *f*(α) = 1 − α is unsuitable for LGD material, the dynamic mode function needing to be re-selected. Table A1 in Appendix A shows the first iteration results of *E***_a_** and *R*^2^ using the trial and error method under different dynamic mode functions. It can be obtained from the attached Table A1 that *f*(α) = 1/2 × (1 − α)[−ln(1 − α)]^−1^, whose integral form is *G*(α) = [−ln(1 − α)]^2^, has the best linearity, so it can be chosen as a dynamic model function of LGD. Table 9 shows the iteration results of LGD using the Bagchi methods when the function of *f*(α) = 1/2 × (1 − α)[−ln(1 − α)]^−1^ is applied. The activation energies of step1 is 91.13 kJ/mol and step2 is 193.12 kJ/mol for the primary sample. After 5000 h aging test, the decline is 3.58% and 1.70% individually for step1 and step2. Figure 6 shows the plot of $\ln[\frac{d\alpha /dT}{f(\alpha )[\frac{E(T-T_{0})}{RT^{2}}+1]}]$ verses 1/*T* for the thermal degradation of D33, E44 and LGD samples. The results of the composites have a good linear relationship in Figure 6.

From the view of chemical activation energy calculated using Bagchi equation, the activation energy required for thermal decomposition of D33 material is the highest, and *E*_a_ of E44 composite is the lowest, which is consistent with the basic thermogravimetric analysis. After 5000 h aging test, the activation energies of the three materials decrease, and the activation energy of LGD drops to the lowest which means that its aging resistance performance is the best. By contrast, E44 materials have the lowest absolute value of activation energy (much less than that of D33 and LGD samples). After the aging test, E44 materials have the biggest decline of *E*_a_, showing the worst thermal stability.

● Integral method

Table 10 and Table 11 show activation energy of the samples calculated by Broido methods and Coats–Redfern methods respectively.

As can be seen from Table 10 and Table 11, D33 samples suffer smaller declines (3.01% for Broido methods, 3.27% for Coats-Redfern methods). However, for E44, this number is greater (38.57% for Broido methods, 41.99% for Coats–Redfern methods). The activation energy of aged E44 materials is much lower than that of other materials. After the aging test, thermal degradation activation energy of the LGD material increases. It is contradictory to the basic thermogravimetric analysis and the reaction kinetics analysis using the differential method. This paper argues that it is due to the inaccurate result caused by the integral method.

**Figure 7:**
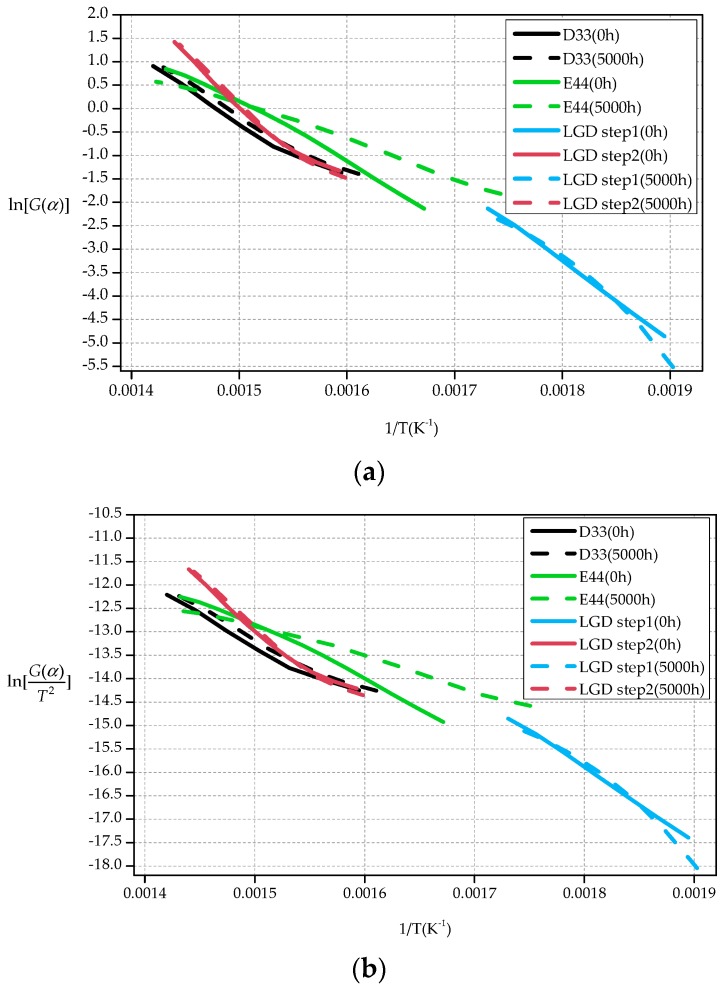
Plot for determination of *E*. (**a**) Broido plot; (**b**) Coats–Redfern plot.

Figure 7a is the plot of ln[G(α)] verses 1/T for the thermal degradation of D33, E44 and LGD samples. Figure 7b is the plot of $\ln[\frac{G(\alpha )}{T^{2}}]$ verses 1/T for the thermal degradation of these materials.

As can be seen from Figure 7, using the selected reaction mechanism function *f*(α), the data calculated by Broido methods and Coats-Redfern methods has good linear relationships, indicating that the selected reaction mechanism function *f*(α) is appropriate.

### 3.3. Surface Morphology

**Figure 8:**
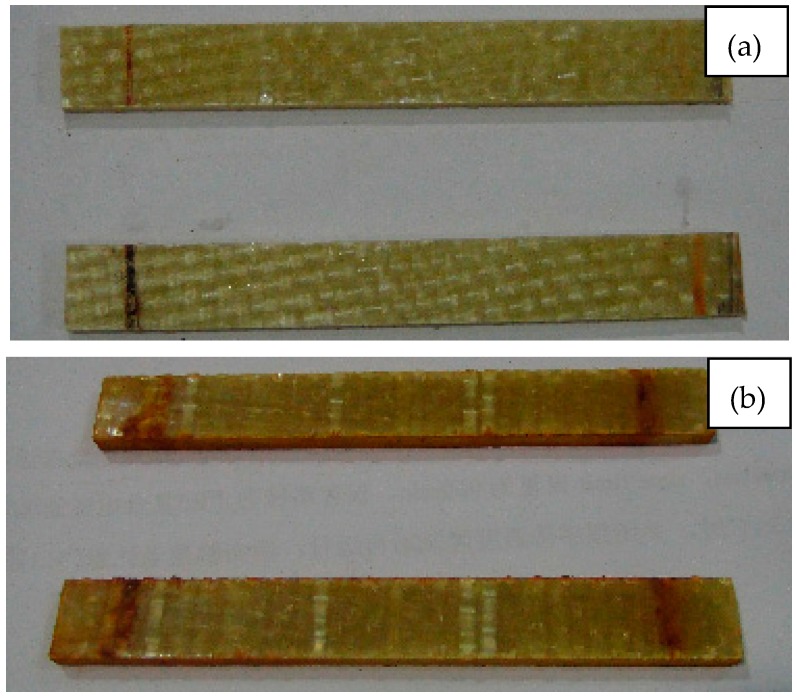
The surface image of D33 composite samples at (**a**) 0 h and (**b**) 5000 h.

**Figure 9:**
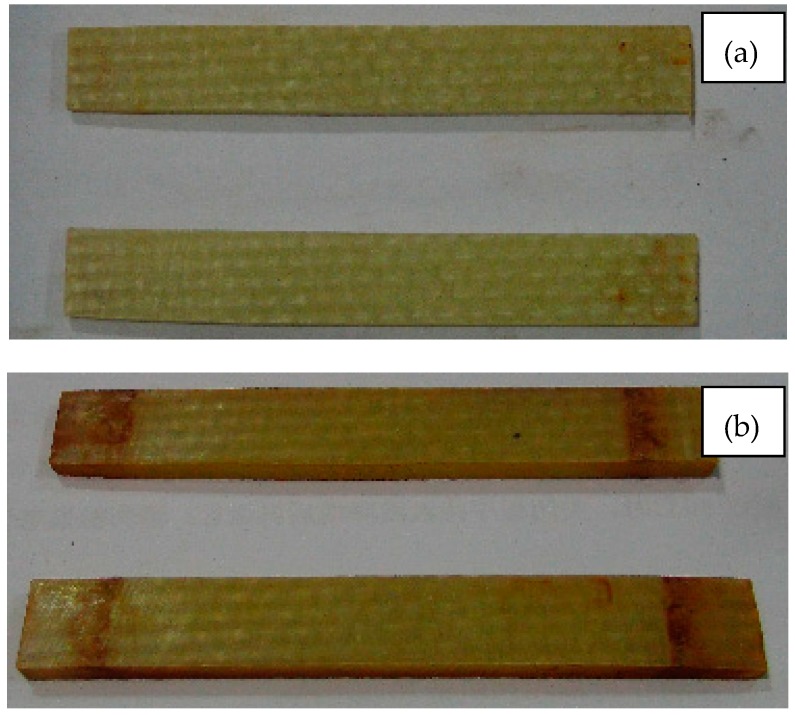
The surface image of E44 composite samples at (**a**) 0 h and (**b**) 5000 h.

**Figure 10:**
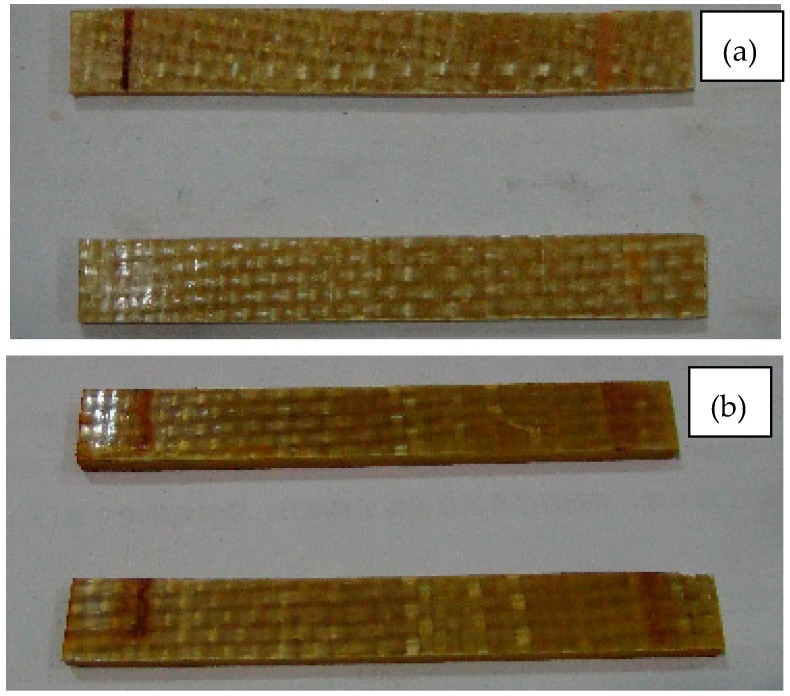
The surface image of LGD composite samples at (**a**) 0 h and (**b**) 5000 h.

Macroscopically, the color change of the composite material can reflect the aging degree, because the surface of resin matrix composite will lose luster during long-term using process. Figure 8, Figure 9 and Figure 10 are surface photos of the three kinds of composites before and after the aging test. From the observation of the specimens, specimens in Figure 8b, Figure 9b and Figure 10b are significantly darker than these in (a). The dividing lines which represent the interface between resin and fiber can be clearly seen in Figure 8b, Figure 9b and Figure 10b, disclosing the destruction of interface properties in the aging process.

**Figure 11:**
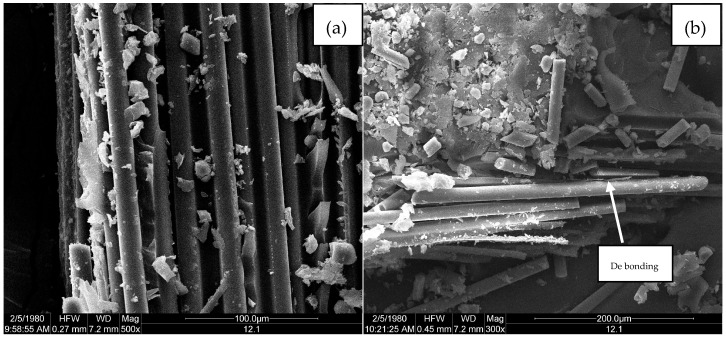
FESEM micrographs of D33 at (**a**) 0 h and (**b**) 5000 h.

**Figure 12:**
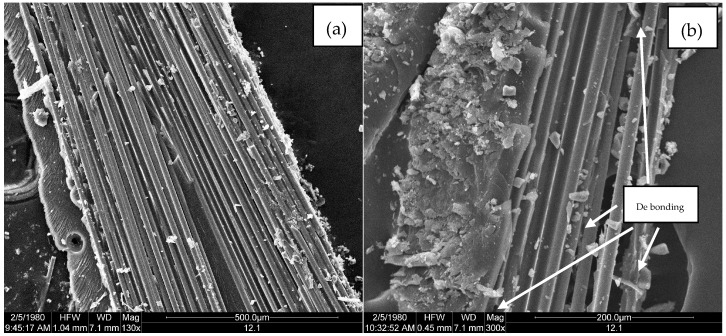
FESEM micrographs of E44 at (**a**) 0 h and (**b**) 5000 h.

**Figure 13:**
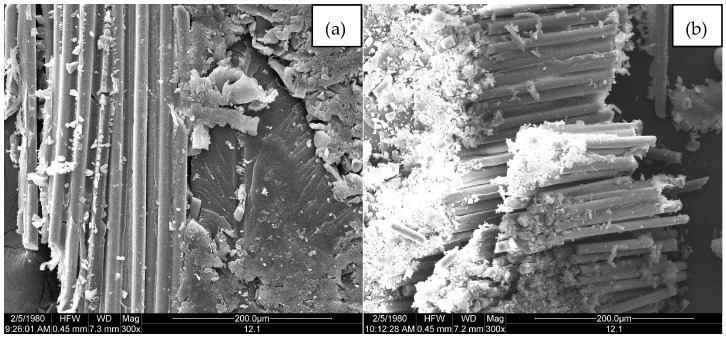
FESEM micrographs of LGD at (**a**) 0 h and (**b**) 5000 h.

Figure 11, Figure 12 and Figure 13 are microscopic morphology images of specimens magnified hundreds of times by FESEM. In the process of destroying the specimen, the matrixes produce a lot of debris. It can be seen from Figure 11, Figure 12 and Figure 13 that there are no erosions. Figure 11a, Figure 12a and Figure 13a show that the fibers are wrapped tightly by resin, which indicates that the fiber and the matrix have good bonding properties. As shown in Figure 11b and Figure 13b, the failure mode of the fiber is mainly fiber breakage, and the cross section is flat. It can be observed from Figure 11b and Figure 13b that the resin around the fibers shows an insignificant decrease compared with Figure 11a and Figure 13a, indicating that the fiber-resin interface properties of LGD and D33 specimens are not greatly reduced. However, the resin around the fiber is remarkably reduced in Figure 12b, which means the bonding property between the fiber and the matrix of E44 composite material decreases in the progress of aging. There are two main reasons for the interfacial damage of the composites: (1) The immersion of the water causes a shear stress at the fiber/matrix interface; (2) Chemical substances immerse inside the substrate react chemically with polar groups on the glass fiber and fiber/matrix interface, resulting in interfacial degradation.

## 4. Discussion

Just as *Difference between the tracking and erosion and accelerated aging tests on polymeric insulators* in IEC/TR 62730-2012 [13] says: “Although this Technical Report describes several tracking and erosion tests which often may be referred to in the literature as ‘aging tests’, it is important to note that they are not accelerated aging tests in the sense that these tests do not simulate exactly real life degradation conditions nor do they accelerate them to give a life-equivalent test in a short time. Rather, they use continuous, cyclic or combined stresses to try to detect potential weaknesses which could compromise the insulators performance in service. The tests are better described as screening tests, which can be used to reject materials, designs, or combinations thereof which are inadequate.” In accordance with the IEC test procedure, if the composite material has no obvious defects after 5000 h test, it can be used for the production of transmission towers. In this paper, there were no clear defects for these three materials, which means that this indicator meets the acceptance criteria of relevant design criteria [13,27,28].

● Bending Test

In this test, although the bending strengths of the three materials decreased after 5000 h, absolute values were greater than 400 MPa, which fully meets the requirements of the design criteria (380 MPa for Q420 steel if the thickness of the steel <16 mm [27,28]). The design code does not specify the value of bending modulus. The *E***_b_** of the three composite materials was about one tenth of that of the steel (the bending modulus of steel is 206 GPa), which means the composite tower will have a greater deformation on the same loading condition. Therefore, structural deformation control must be focused on during the design process of the composite tower [29]. 

There are no relevant criteria for bending modulus and bending strength retention rates. In IEC 62217-2012 [30], the acceptance criteria of hardness test is that the hardness of each specimen shall not exceed 20% from the pre-boiled value. Reference [31] has regarded 50% strength retention rate as an indicator of the end life of glass fiber reinforced polymer in transmission towers. After the 5000 h aging test, the bending modulus and strength retention rates of the composite samples were higher than 90%, which was far from the end of life.

● Thermogravimetry Analysis

In the case of thermal stability, there is also no specific requirement in design criteria. Likewise, this paper regards 50% thermal stability degradation as an indicator of the end of life. The rate of activation energy decline was slow and less than 10% during the aging test for D33 and LGD materials. However, the activation energy of E44 material decreased by 25.04%. All these three materials did not reach the end of life. In addition, anti-aging performance of E44 was not as good as D33 and LGD materials.

● Morphology Test

The IEC/TR 62730-2012 acceptance criteria of 5000 h test at multiple stresses for composite insulator says: “the test is regarded as passed if, on both test specimens: no tracking occurs; for composite insulators: erosion depth is less than 3 mm and does not reach the core, if applicable; for resin insulators: erosion depth is less than 3 mm; no shed, housing or interface is punctured.” As can be seen from the acceptance criteria, the surface and microscopic morphology are important factors to assess whether the material could pass the 5000 h test. The sample that has good surface morphology and no corrosion can be considered having passed the aging test. In this paper, the surfaces of the three materials had not significantly punctured, and the resins around the fibers did not show an insignificant decrease for LGD and D33 materials, indicating that LGD and D33 materials meet the aging requirements according to the IEC/TR 62730-2012. However, the resin around the fiber was remarkably reduced for E44 materials.

## 5. Conclusions

Bending modulus of the three materials decreased with the multi-factor aging time from 0 h to 5000 h, declining by about 6% at 5000 h. The *E*_b_ of the LGD composite samples was about 2 GPa higher than that of the other two materials during the whole aging process. The bending modulus retention rates of the three composite samples were higher than 94% after the 5000 h aging test, and the mechanical properties and aging resistance of these composites are good. The aging test ruined the thermal stability of D33, E44 and LGD composites. D33 samples have the best thermal stability, and the E44 samples have the worst. The activation energy of these composites decreased, and the activation energy of the D33 material was the highest. The most probable mechanism functions of D33, E44 and LGD materials are *f*(α) = 1 − α, *f*(α) = 1 − α and *f*(α) = 1/2 × (1 − α)[−ln(1 − α)]^−1^ respectively. During the aging process, the interface properties of these materials were destroyed as observed by FESEM. After the aging test, for E44 specimen the resin wrapped around the fiber gradually fell off, while the LGD and D33 samples maintained a good microstructure.

On the whole, D33 and LGD composite materials have excellent aging resistance and thermal stability, and have passed 5000 h multi-factor accelerated aging test recommended by IEC. These two composites can be used as main ingredients for transmission composite towers and crossarms.

## Figures and Tables

**Table 1:** The specific information of the selected materials.

Sample Name	LGD	E44	D33
resin matrix	modified polyurethane resin	epoxy resin	modified unsaturated polyester resin
manufacturer	NARI (Wuhan, China)	Baling Petrochemical Co. Ltd. (Yueyang, China)	Ashland (Changzhou, China) Chemical Co. Ltd.
Resin’s specification	Self-developed by NARI	E-44 is 6101 epoxy resin, which is a general purpose resin	D33 is 197# unsaturated polyester resin, which is a general purpose resin
Specimen capacity	12	12	12
Resin’s Synthesis Method	By introducing propoxylated bisphenol A into unsaturated polyester segments, the hydroxy-terminated unsaturated polyester are synthesized; then the saturated polyester segments and the polyurethane segments are joined by chemical covalent bonds.	The conventional method is by the condensation reaction of BPA and ECH.	Polyester is synthesized from bisphenol A derivative and maleic anhydride. And then unsaturated polyester resin is made by polyester and styrene.

Notes: In papers published by our research group, each sample has multiple names: LGD being called PU; D33 being called PUR, E44 being called E51 due to the value of epoxy.

**Table 2:** Composite material samples multi-factors aging schedule.

Humidification						**●**										**●**									
Heating				**●**		**●**								**●**		**●**								**●**	
Cooling										**●**										**●**					
Rain		**●**																							
Salt fog								**●**		**●**								**●**		**●**					
Ultraviolet ray		**●**		**●**		**●**								**●**		**●**		**●**		**●**					
Electric field		**●**		**●**		**●**		**●**		**●**		**●**		**●**		**●**		**●**		**●**		**●**		**●**	
Time (hours)	0		2		4		6		8		10		12		14		16		18		20		22		24

“**●**” indicates that the aging factor is added at this time.

**Table 3:** Results of bending modulus of composites during the aging test.

Time	D33		E44		LGD	
	*E*_b_/GPa	*Y*/%	*E*_b_/GPa	*Y*/%	*E*_b_/GPa	*Y*/%
0	16.43	100.0	16.24	100.0	18.38	100.0
500	15.24	92.8	15.54	95.6	17.00	92.5
1000	15.38	93.6	15.41	94.8	16.93	92.1
1500	15.33	93.3	14.73	91.3	17.51	95.3
2000	15.57	94.8	16.00	98.5	17.49	95.2
2500	15.39	93.7	15.38	94.7	17.15	93.3
3000	15.38	93.6	15.86	97.7	17.31	94.2
4000	15.45	94.1	15.44	95.1	17.20	93.6
5000	15.45	94.1	15.27	94.0	17.32	94.2

**Table 4:** Results of bending modulus of resin matrixes during the aging test.

Time	LGD		E44		D33	
	*E*_b_/GPa	*Y*/%	*E*_b_/GPa	*Y*/%	*E*_b_/GPa	*Y*/%
0	2.58	100.0	2.82	100.0	2.67	100.0
1000	2.80	108.5	2.62	92.9	2.61	97.8
2000	2.65	102.7	2.61	92.5	2.65	99.3
3000	2.65	102.7	2.56	90.8	2.61	97.8
4000	2.68	103.8	2.61	92.6	2.64	98.9
5000	2.63	101.9	2.60	92.2	2.64	98.9

**Table 5:** Results of bending strength (*f*) of composites before and after the aging test.

Time	D33		E44		LGD	
	*f*/MPa	*Y*/%	*f*/MPa	*Y*/%	*f*/MPa	*Y*/%
0	415.0	100.0	437.0	100.0	428.4	100.0
5000	401.5	96.7	405.5	92.8	419.5	97.9

**Table 6:** Basis data of the composites through TGA.

Matrrials	Ageing time/h	OT (°C)	DTG_max_ (°C)	*T*_5%_ (°C)	FR (%)
D33	0	354	401	278	39.0
	5000	348	400	297	55.0
E44	0	325	365	324	53.4
	5000	290	352	298	64.6
LGD	0	247	405	263	53.3
	5000	243	394	259	44.5

Notes: OT is the onset degradation temperature; DTG_max_ is the DTA peaks temperature; *T*_5%_ is the 5% loss of mass temperature; FR is the final residue.

**Table 7:** The determined value of *T*_0_ at α which is small.

Materials	D33		E44		LGD1	
	0 h	5000 h	0 h	5000 h	0 h	5000 h
α	0.015	0.021	0.022	0.018	0.018	0.014
*T*_0_ (K)	364.8	365.6	514.9	441.0	494.5	508.9

**Table 8:** The iteration results of D33 and E44 using the Bagchi methods when *f*(α) = 1 − α.

	Materials	D33				E44			
		0		5000		0		5000	
Iteration		*E*_a_ (kJ/mol)	*R*^2^	*E*_a_ (kJ/mol)	*R*^2^	*E*_a_ (kJ/mol)	*R*^2^	*E*_a_ (kJ/mol)	*R*^2^
1		134.49	0.9880	124.53	0.9774	74.16	0.9795	54.66	0.8923
2		134.35	0.9880	124.38	0.9774	72.68	0.9794	54.53	0.8920
3		134.35	0.9880	124.38	0.9774	72.75	0.9794	54.53	0.8920
4						72.75	0.9794		

**Table 9:** The iteration results of LGD using the Bagchi methods when *f*(α) = 1/2 × (1 − α)[−ln(1 − α)]^−1^.

	Materials	LGD 0 h				LGD 5000 h			
		Step1		Step2		Step1		Step2	
Iteration		*E*_a_ (kJ/mol)	*R*^2^	*E*_a_ (kJ/mol)	*R*^2^	*E*_a_ (kJ/mol)	*R*^2^	*E*_a_ (kJ/mol)	*R*^2^
1		96.39	0.9660	195.37	0.9977	97.93	0.9137	192.67	0.9912
2		90.67	0.9643	193.11	0.9977	85.89	0.9094	189.81	0.9911
3		91.17	0.9645	193.37	0.9976	88.30	0.9099	189.83	0.9911
4		91.12	0.9645	193.12	0.9976	87.79	0.9098	189.83	0.9911
5		91.13	0.9645	193.12	0.9976	87.90	0.9098		
6						87.87	0.9098		
7						87.87	0.9098		

**Table 10:** Broido methods *E* value of the sample for the thermal degradation.

Materials	Ageing time/h	*E*_a_ (kJ/mol)	*R*^2^	*f*(α)
D33	0	111.52	0.9821	*f*(α) = 1 − α
	5000	108.16	0.9837	
E44	0	105.00	0.9947	
	5000	64.50	0.9962	
LGD step1	0	130.28	0.9880	*f*(α) = 1/2 × (1 − α)[−ln(1−α)]^−1^
	5000	146.09	0.9582	
LGD step2	0	153.28	0.9736	
	5000	160.30	0.9794	

**Table 11:** Coats–Redfern methods *E* value of the sample for the thermal degradation.

Materials	Aging time/h	*E*_a_ (kJ/mol)	*R*^2^	*f*(α)
D33	0	100.50	0.9786	*f*(α) = 1 − α
	5000	97.21	0.9805	
E44	0	94.26	0.9929	
	5000	54.68	0.9913	
LGD step1	0	121.04	0.9859	*f*(α) = 1/2 × (1 − α)[−ln(1 − α)]^−1^
	5000	136.88	0.9522	
LGD step2	0	142.30	0.9699	
	5000	149.36	0.9766	

## Appendix A

**Table A1:** The first iteration results using the trial and error method under different dynamic mode functions.

	Materials	LGD 0 h				LGD 5000 h			
		Step1		Step2		Step1		Step2	
*f*(α)		*E*_a_ (kJ/mol)	*R*^2^	*E*_a_ (kJ/mol)	*R*^2^	*E*_a_ (kJ/mol)	*R*^2^	*E*_a_ (kJ/mol)	*R*^2^
*f*(α) = 1/2 × (1 − α)[−ln(1 − α)]^−1^		96.39	0.9660	195.37	0.9977	97.93	0.9137	192.67	0.9912
*f*(α) = 2/3 × (1 − α)[−ln(1 − α)]^−1/2^		61.40	0.9267	157.05	0.9937	49.34	0.8451	152.59	0.9801
*f*(α) = 1/3 × (1 − α)[−ln(1 − α)]^−2^		166.37	0.9861	272.01	0.9974	150.48	0.8685	272.82	0.9966
*f*(α) = (1 − α)^1/2^[1 − (1 − α)1/2]^−1^		88.67	0.9517	137.40	0.9644	89.24	0.8913	134.39	0.9350
*f*(α) = 3/2 × [(1 − α)^−1/3^ − 1]^−1^		85.56	0.9497	144.42	0.9851	88.25	0.8883	144.01	0.9623
*f*(α) = 1 − α		-	-	-	-	-	-	-	-

Notes: “-” indicates that the result of the first step is non-linear using the current *f*(α). The results obtained by these following function, including *f*(α) = α^−1^/2, *f*(α) = 4(1 − α)^1/2^[1 − (1 − α)1/2]^1/2^, *f*(α) = α(1 − α), *f*(α) = 4α^3/4^, *f*(α) = 3α^2/3^, *f*(α) = 2α^1/2^, *f*(α) = 1, *f*(α) = 1/2 × α^−1^, *f*(α) = 2/3 × α^−1/2^, *f*(α) = 4(1 − α)^3/4^, *f*(α) = 3(1 − α)^2/3^, *f*(α) = (1 − α)^2/3^, *f*(α) = (1 − α)^1/2^, *f*(α) = 1/2 × (1 − α)^−1^, *f*(α) = 1/3 × (1 − α)^−2^, *f*(α) = 1/4 × (1 − α)^−3^, *f*(α) = (1 − α)^2^, *f*(α) = 2 × (1 − α)^3/2^, *f*(α) = α, *f*(α) = 1/2 × (1 − α)^3^, are also nonlinear.
